# Small Molecules with Spiro-Conjugated Cycles: Advances in Synthesis and Applications

**DOI:** 10.3390/ijms242417584

**Published:** 2023-12-18

**Authors:** Elena K. Beloglazkina

**Affiliations:** Department of Chemistry, M.V. Lomonosov Moscow State University Leninskie Gory, 1-3, 119991 Moscow, Russia; beloglazki@mail.ru

Dear Colleagues,

The purpose of this Special Issue was to attract the attention of researchers to the features of the synthesis and physico-chemical and biological investigation of spiro-conjugated organic compounds and to show the possibilities and advantages that the introduction of a spiro-conjugated fragment into small organic molecules provides. One of these advantages is the development of new experimental methods in organic chemistry, which can be easily transferred to the preparation of molecules of other structural types and may prove useful in the development of new effective synthetic methodologies.

The second most important feature of spiro-jointed organic molecules is the unique chemical, physical, and biological properties that the resulting spiro-compounds can possess. Among these advantages is, first of all, high biological activity. It is known that the effectiveness of the interaction between drug molecules and a biotarget is directly related to the side effects of drugs. This means that the higher the selectivity of the molecule with respect to the active center, the fewer body functions that are not targeted for treatment will be affected. The most important factor in the development of drugs is the limitation of the conformational mobility of the synthesized molecules, which makes it possible to fix the required spatial position of important substituents that bind to biological targets. From this point of view, molecules containing a spiro junction of two or more carbo- or heterocycles are of significant interest. The rigidity of spiro junctions in molecular frameworks makes it possible to fix the required spatial arrangement of exocyclic substituents, which are important for interaction with biological targets and the properties of developed materials. The correct selection of substituents in molecules containing the same pharmacophore fragment allows fine tuning of the compound structures, making it possible to use them in drugs with different types of actions. For example, molecules containing spiro-membered heterocyclic fragments are capable of exerting a selective effect on AKT1 kinase through the PI3K pathway [1], on the protein-protein interaction of p53-MDM2 [2,3], and so forth.

Due to their wide range of prominent physiological activities, heterocyclic spiro-compounds have always been among the most attractive and privileged organic scaffolds in modern medicinal chemistry [4,5]. For example, spiro-jointed nitrogen-containing heterocyclic derivatives have emerged as attractive synthetic templates because of their prevalence in a significant number of natural-like products [6]. Some alkaloids containing spiro-motifs were first isolated from plants of the *Apocynaceae* and *Rubiacae* families [7]. The basic structural feature of this type of compound is the spiro point at position 3 of the oxindole fragment. This joint can be formed by the attachment of heterocyclic motifs, thereby providing a significant degree of diversity. As a result, spiro-oxindoles are reasonably regarded as appropriate templates for drug design and development. They can also be readily used as convenient starting points or intermediates in the synthesis of a wide range of structurally diverse natural-like products.

The design and development of novel potent anticancer therapeutics are the most important tasks of synthetic organic and medicinal chemistry. Among the compounds with antitumor action, an important place is occupied by the spiro and dispiro derivatives of indolinones, because the indolinone fragment simulates the tryptophan moiety and is in many cases involved in interactions with biological targets [8,9,10,11]. Hence, spiro-oxindole alkaloids have shown significant anticancer activity [4,12,13]. Several manuscripts [14,15] support the potential of spiro-linked indolinones as highly cytotoxic as well as antibacterial active compounds. Some spiro-indolinones may be also a poliovirus and human rhinovirus 3C-proteinase inhibitors [16]. Significant cytotoxic activity with high selectivity was also found for other heterocyclic derivatives with a spiro junction [17,18].

The scope of biological activity of spiro-linked small molecules is, however, not limited to antitumor activity. Thus, the potential of spiro heterocyclic compounds to treat Alzheimer disease was proven in the work of T. Ben Hadda et al. [19] The manuscript by B. Bennani et al. [20] gives an idea to use some di-substituted-4′H-spiro [isothiochromene-3,5′-isoxazol]-4(1H)-ones as potential drugs against *Mycobacterium tuberculosis* and HIV-1 inhibitors.

The interesting charge-transport properties of molecules with a spiro junction are demonstrated by the work of U. Bach et al. [21], describing a family of spiro-conjugates carbocycles combining the high morphological stability with commonly only observed in polymeric systems with the high charge mobility of low molecular weight charge transport materials.

Organic spiro derivatives can also be used as photochromic molecules. Thus, in the article by J.-M.A. Castán and co-authors [22] the interesting photophysical properties of the spiro-indoline naphthoxazines and naphthopyrans were described; such molecules demonstrate an acidochromic behavior and lead to the formation of protonated merocyanines absorbing in the visible range under acidic conditions. When the compounds are studied in acidic conditions under illumination, they show different behaviors. The spiro-indoline naphthoxazines keep a positive photochromism while the spiro-indoline naphthopyrans show a negative photochromism.

It is also very important that spiro-linked carbo- and heterocyclic compounds can be obtained by various types of cyclization reactions with high regio- and stereoselectivity and atomic precision. Such reactions are atom-economical and make it possible to significantly complicate the molecular structure in one synthetic step. From the point of view of synthetic organic chemistry, I also cannot help but note that spiro-jointed derivatives are very attractive molecules from the point of view of the green chemistry paradigm, since their syntheses by concerted addition reactions require a minimum number of synthetic steps and all atoms of the initial compounds are included in the target products without the formation of additional reaction products. In this case, the targeted synthetic steps can be carried out with high stereoselectivity [23,24].

It is our earnest hope that this Special Issue will provide some insight into the current state of research into the synthesis and practical application of small organic molecules containing the spiro-junction moiety and will assist in ongoing efforts to more efficiently develop and potentially utilize such molecules in chemistry, physics, and biology.
